# *Iris* × *germanica* L. and *Nephrolepis exaltata* (L.) Schott, two novel strontium hyperaccumulators

**DOI:** 10.1038/s41598-026-57010-6

**Published:** 2026-06-08

**Authors:** Popovici Patriciu, Claudia Maria Gutu, Robert Viorel Ancuceanu, Irina Zarafu, Adriana Urda, Andrei Tiberiu Cucuruz, Maria Cojocaru-Toma, Mihaela Dinu

**Affiliations:** 1 Department of Pharmaceutical Botany and Cell Biology, Faculty of Pharmacy, Carol Davila University of Medicine and Pharmacy, Bucharest, Romania; 2 Department of Toxicology, Faculty of Pharmacy, Carol Davila University of Medicine and Pharmacy, Bucharest, Romania; 3 Inorganic, Organic Chemistry, Biochemistry and Catalysis Department, University of Bucharest, Bucharest, Romania; 4 Faculty of Chemical Engineering and Biotechnology, National University of Science and Technology Politehnica Bucharest, Bucharest, Romania; 5 Faculty of Pharmacy, Nicolae Testemițanu University of Medicine and Pharmacy, Chișinău, Moldova

**Keywords:** Phytoremediation, *Nephrolepis exaltata* (L.) Schott, *Iris* × *germanica* L., Vanadium, Strontium, Ecology, Ecology, Environmental sciences, Plant sciences

## Abstract

We examined the uptake and the effects of strontium (Sr) and vanadium (V) on the growth of hydroponically cultivated species *Nephrolepis exaltata* (L.) Schott (NE) and *Iris* × *germanica* L. (IG). Each species was cultivated in deep water culture tanks in solutions containing 0–80 mg/L NH₄VO₃ and subsequently 50–200 mg/L Sr(NO₃)₂ for two weeks. The accumulated ions in the plant parts were determined using inductively coupled plasma mass spectrometry (ICP-MS). The results showed that across tested concentrations, neither strontium nor vanadium exerted any sizable effect on the growth of the two species. Both species efficiently accumulated Sr, with translocation factors (TF) consistently above 1 (approximately 1.2–2.2) and bioconcentration factors (BCF) ranging from approximately 6 to 9, meeting the criteria for classification as Sr hyperaccumulators. Total metal uptake reached values on the order of 10^3^ mg/kg in both species at the highest concentrations tested. These results suggest that both NE and IG hold significant promise for Sr phytoremediation applications, demonstrating high tolerance to V and substantial root retention capacity, suggesting their potential utility in the phytostabilization of V-contaminated soils.

**Supplementary Information:**

The online version contains supplementary material available at 10.1038/s41598-026-57010-6.

## Introduction

The cleanup of toxic metal-contaminated soils or waters can be achieved economically by means of phytoremediation, which uses plants to extract, accumulate, or immobilize contaminating metals from the soil or water. Phytoextraction is the process by which plants remove toxic metals from the soil; rhizofiltration describes the removal of toxic metals from waters with the help of plants; phytostabilization is the process by which plants reduce the bioavailability of toxic metals in the soil^1^. Most plants that do not hyperaccumulate these metals retain them in the roots, where they complex and deposit them in vacuoles. Some plants do not allow the uptake of toxic metals from the environment, complexing them with exudates, or isolating them in cell walls^2^.

Vanadium (V) is a transition metal that is widely distributed in the environment and is primarily associated with industrial activities. At low concentrations, it may stimulate plant growth, but at higher concentrations, especially in its pentavalent form, it induces oxidative stress, disrupts photosynthesis, and inhibits growth^3,4^. V exhibits numerous toxic effects on the human body^5,6^. Most plants retain V predominantly in their roots, limiting its translocation. Several species have been investigated for V phytoremediation, although their efficiency is often constrained by toxicity effects or low translocation capacity^4,7–9^.

Strontium (Sr) is an alkaline-earth metal that can be introduced into the environment through natural occurrences and human activities, such as mining and nuclear operations. Because it shares chemical properties with Ca, Sr has the potential to disrupt plant physiological functions, resulting in inhibited growth and oxidative stress^10–12^. Its radioactive isotope, strontium-90, is of particular environmental concern because of its persistence and bioaccumulation potential^11^. It the human body, at high doses, strontium replaces calcium and can cause serious health damage^13^.

*Nephrolepis exaltata* (L.) Schott, NE, called the Boston or sword fern, is a tropical perennial fern originating from the Americas, which is now widely distributed worldwide and is very common as an ornamental houseplant^14^. In the scientific literature, the therapeutic use of this fern is the traditional treatment (in the island of Fiji) of women’s menstrual disorders^15^. NE has been studied for its phytoremediation properties of manganese, zinc, and copper in soils^16^, arsenic accumulation^17^, lead and cadmium, bioaccumulation, and physiological effects of mercury on its growth^18^.

*Iris x germanica* L., IG, the bearded iris, is a medicinal plant with a long tradition of medicinal use, widely cultivated for ornamental purposes and the most investigated species of the genus *Iris*^23^. Its phytoremediation potential has been studied for lead^24^ and chromium^25^.

Neither *Nephrolepis exaltata* (L.) Schott, NE, nor *Iris* × *germanica* L., IG, two medicinal species at different levels of evolutionary development, a pteridophyte and a monocot having the same underground morphology, the rhizome, have been tested for their phytoremediation potential of these toxic metals (Sr and V). Besides, the toxic effects of these metals on the two plant species when grown in hydroponic medium are unknown. In addition, the hydroponic medium offers better possibilities for evaluating these aspects, as it eliminates the confounding effect of soil characteristics and soil microbiome in geoponic cultivation^26^. The hydroponic medium also avoids mass transfer (bioavailability) problems that characterize experiments carried out in a geoponic system^27^.

Despite the growing interest in phytoremediation, no study has systematically evaluated the tolerance, accumulation, and translocation characteristics of these two species (from different evolutionary groups, a pteridophyte and a monocot) when exposed to Sr and V, especially in controlled hydroponic environments. These systems offer improved control over metal bioavailability by removing soil-related variations. Therefore, the objectives of this study were as follows:

i. To assess the tolerance of NE and IG to Sr and V exposure;
ii. To evaluate the effects of low metal concentrations on plant growth;
iii. To determine the distribution of metals between roots, rhizomes, and leaves;
iv. To calculate translocation factors (TF), bioconcentration factors (BCF), and total metal uptake (TME); and
v. To assess the suitability of these species for phytoextraction or phytostabilization.

## Materials and methods

### Plant material and growth conditions

NE was commercially purchased from a commercial vendor (Dedeman srl, country of origin: Holland), and IG was obtained courtesy of the Botanical Garden „Dimitrie Brândză”, Bucharest. Because the original growth substrate of the plants at the time of purchase is unknown, baseline residual metals may lead to a slight underestimation of the calculated accumulation factors, particularly in low-dose treatments, where background levels represent a larger proportion of the total measured concentrations. This likely explains the detectable concentrations of Sr and V in the control plants, which reflect residual internal metal pools rather than uptake during the experimental period.

The deep-water culture system used was custom-built, rather than commercially purchased. Each tank was made of 25 L opaque PVC boxes, six 12 cm mesh pots, and expanded clay, and each tank was filled with a 15 L solution made with tap water and 30–40 ml of concentrated liquid fertilizer for the vegetative growth cycle of green plants (SC Amia International). The mineral solution used was similar to that of the Flora series from General Hydroponics. The solution was constantly aerated using a Hailea ACO 9602 air pump with a flow rate of 7.2 L of air per minute. The tubing was 4 mm in diameter, and the 150 mm air stones were ceramic. The concentrated mineral solution contained the following dissolved minerals (mg/ml): 60 N, 40 P_2_O_5_, 50 K_2_O, 0.21 FeEDTA, 0.13 B, 0.03 Cu, 0.11 Mn, 0.011 Mo, and 0.058 Zn. The day/night light cycle was 12 h/12 h, the lights were 250 W metal halide GIB growth spectrum lights, and the photosynthetic active radiation measured in μmol/m^2^s and its standard deviation for each tank were: tank 1–47.33 and 11.16; tank 2–56.50 and 11.74; tank 3–56.83 and 4.91; tank 4–52.33 and 5.35.

Six specimens of each species were used in each tank, and each tank represented one experimental unit corresponding to a single treatment concentration. For each species and metal, four tanks were used: three treatment tanks (different concentrations) and one control tank. Thus, each treatment was represented by a tank containing six biological specimens. As the aim of this study was an initial screening of metal tolerance, accumulation, and translocation under controlled hydroponic conditions, the experiments were performed once for each treatment condition. Statistical analyses were therefore conducted using measurements obtained from individual plants within each tank, while accounting for potential within-tank dependence where appropriate through mixed-effects modeling (tank/pot structure treated as a random effect when feasible).

Ammonium metavanadate, NH_4_VO_3_ (Merck), was used at the following concentrations: none for the control; 10, 20, and 40 mg/L for IG to determine the potential growth benefits of these low concentrations and 20, 40, and 80 mg/L for NE to determine the potential toxicity of these high concentrations. Strontium nitrate, Sr(NO₃)₂ (Merck), was used at the following concentrations: 50 mg/L, 100 mg/L, 200 mg/L, and none for the control. The atmospheric cultivation conditions and nutrient solution values for NE are shown in Tables S1–S4 and those for IG are shown in Tables S5–S8. Different vanadium concentration ranges were selected for the two species based on preliminary observations and literature data suggesting higher vanadium tolerance in ferns than in many angiosperms, as well as to allow exploration of distinct biological questions. For IG, lower concentrations (10–40 mg/L) were selected to investigate the potential hormetic or growth-promoting effects reported at low V exposure levels, whereas for NE, higher concentrations (20–80 mg/L) were used to evaluate tolerance limits and potential toxicity under more severe exposure conditions.

During cultivation, the nutrient solution parameters were monitored regularly. Across all experiments, the average solution pH remained within the range of 4.34–6.31, average total dissolved solids within 447–671.7 ppm, and an average solution temperature within 22.72–28.85 °C (full measurements are reported in Supplementary Tables S5–S8).

When harvested, leaf length and number were recorded. The plants were then dried and separated into leaves and rhizomes (and roots for IG), which were weighed. The plant material was brought to a constant weight using an oven at 105 °C and measured on analytical scales (Partner PS 600/C/2). The analyses were performed using inductively coupled plasma mass spectrometry for IG and NE, simultaneously for both V and Sr mineralized using a microwave-assisted protocol.

### Statistical analysis

All statistical analyses were performed using R, version 4.4.1^28^. Where possible (multiple measurements on a single specimen from the same pot), the use of a mixed-effects model was explored (R package “lme4”^29^ or, as appropriate, “robustlmm”^30^); the growing medium was treated as a fixed variable and the pot as a random variable. For the other situations we used, as appropriate, parametric models (Welch t-test, linear regression models equivalent to univariate ANOVA—in the case of a categorical independent variable) and robust models (Yuen test for means test and a bootstrap-based variant of it, a t-test based on robust estimators, a robust test for independent groups based on the comparison of medians, a test based on the comparison of arbitrary quantile-based test—R package “WRS2”^31^. Robust regression models—R package “robustbase” package^32^), were used when linear regression assumptions were not satisfied. For evaluation of the assumptions, we used both graphical tools (QQ-plot, histograms of residuals, etc.) and inferential (a variety of tests for linearity, normality, and homoscedasticity—mainly the R packages “car”^33^, “fBasics”^34^, “lmtest”^35^, “PMCMRplus”^36^ and “onewaytests”^37^). Effect sizes were robustly evaluated using the functions akp.eff and onewaytests of the WRS2 R package^37^). Descriptive plots were generated using the R package “ggstatsplus”^38^. The confidence intervals for the correlation coefficients were estimated using bootstrap on 9999 replicates using the “bca” method (R package “confintr”^39^).

## Results

### The effect of vanadium on *Nephrolepis exaltata* (L.) Schott

Following V treatment, N. exaltata accumulated vanadium in both leaves and rhizomes, with concentrations increasing with exposure level (Table 1). A strong positive correlation was observed between the external V concentration and accumulation in leaves (r = 0.93), whereas rhizome accumulation showed greater variability. A slight decrease in rhizome V concentration at the highest treatment level suggests a possible saturation effect.

**Table 1:** Vanadium and strontium content in the vegetative organs of NE measured after ammonium metavanadate exposure.

Sample	Sr (mg/kg)	V (mg/kg)
NE leaves 20 mg/L NH_4_VO_3_	50.1	57.4
NE rhizomes 20 mg/L NH_4_VO_3_	35.1	1304.8
NE leaves 40 mg/L NH_4_VO_3_	58.8	81.6
NE rhizomes 40 mg/L NH_4_VO_3_	48.0	2459.5
NE leaves 80 mg/L NH_4_VO_3_	44.6	105.1
NE rhizomes 80 mg/L NH_4_VO_3_	39.9	1904.8
NE leaves control	53.6	4.1
NE rhizomes control	24.1	15.8

No statistically significant effects of vanadium on growth parameters were observed. Leaf length (Fig. S1) and leaf number (Fig. S2), leaf area (Fig. S3), and biomass-related parameters (Figs. S4–S6) remained unchanged across the treatments (*p* > 0.05). A summary of these results is presented in Table 2.

**Table 2:** Summary of growth parameters (mean ± s.d.), and statistical significance for NE. Means within the same species followed by different letters are significantly different (*p* < 0.05).

Species	Treatment	Leaf length Mean ± s.d	Leaf number Mean ± s.d	Leaf area Mean ± s.d	Dry mass of leaves Mean ± s.d	Dry mass of underground organs Mean ± s.d	Total dry mass Mean ± s.d
NE	Control	25.2 ± 8.4^a^	36.7 ± 12.2^a^	1258.0 ± 273.4^a^	6.3 ± 3.5^a^	2.4 ± 1.5^a^	8.7 ± 4.8^a^
NE	V 10 mg/L	22.6 ± 8.7^a^	35.0 ± 14.3^a^	995.9 ± 441.4^a^	4.4 ± 2.1^a^	1.4 ± 0.6^a^	5.8 ± 2.6^a^
NE	V 20 mg/L	24.8 ± 7.6^a^	39.2 ± 6.4^a^	1322.8 ± 278.3^a^	7.1 ± 2.3^a^	2.9 ± 1.0^a^	10.0 ± 3.2^a^
NE	V 40 mg/L	25.2 ± 7.1^a^	29.5 ± 10.5^a^	1207.1 ± 437.6^a^	5.0 ± 2.3^a^	1.9 ± 1.2^a^	6.9 ± 3.4^a^
NE	Control	25.3 ± 7.8^a^	96.5 ± 38.4^a^	1118.6 ± 329.7^a^	4.4 ± 1.6^a^	1.7 ± 0.6^a^	6.1 ± 1.8^a^
NE	Sr 50 mg/L	23.4 ± 8.5^a^	74.7 ± 22.9^a^	949.4 ± 342.4^a^	3.3 ± 1.8^a^	1.1 ± 0.6^a^	4.4 ± 2.3^a^
NE	Sr 100 mg/L	27.8 ± 8.2^a^	84.6 ± 50.2^a^	1101.5 ± 619.3^a^	3.7 ± 2.5^a^	1.4 ± 0.9^a^	5.1 ± 3.4^a^
NE	Sr 200 mg/L	29.2 ± 7.8^a^	67.0 ± 34.5^a^	955.1 ± 268.1^a^	3.4 ± 0.9^a^	1.3 ± 0.6^a^	4.6 ± 1.5^a^

### The effect of strontium on *Nephrolepis exaltata* (L.) Schott

Strontium accumulated significantly in both leaves and rhizomes, and there was a strong positive correlation with the external concentration (Table 3). Accumulation was more pronounced in the leaves than in the rhizomes. Despite this accumulation, no statistically significant effects on the growth parameters were detected. Leaf length (Fig. S7), leaf number (Fig. S8), leaf area (Fig. S9), and the biomass parameters (Figs. S10–S12) showed no significant differences between the treatments (p > 0.05), although slight decreasing trends were observed at higher concentrations. The results are summarized in Table 2.

**Table 3:** Vanadium and strontium contents in the vegetative organs of NE measured after strontium nitrate treatment.

Sample	Sr (mg/kg)	V (mg/kg)
NE leaves 50 mg/L Sr(NO_3_)_2_	846.6	5.6
NE rhizomes 50 mg/L Sr(NO_3_)_2_	388.0	40.1
NE leaves 100 mg/L Sr(NO_3_)_2_	1321.1	6.9
NE rhizomes 100 mg/L Sr(NO_3_)_2_	664.4	36.2
NE leaves 200 mg/L Sr(NO_3_)_2_	2549.8	9.1
NE rhizomes 200 mg/L Sr(NO_3_)_2_	1784.2	66.7
NE leaves control	51.0	6.0
NE rhizomes control	21.2	46.1

### The effect of vanadium on *Iris × germanica* L. growth

Vanadium accumulation in *I.* × *germanica* was highest in the roots, followed by the rhizomes, with limited translocation to the leaves (Table 4). Accumulation increased with exposure concentration, although the overall levels in the leaves remained low compared to those in *N. exaltata*.

**Table 4:** Vanadium and strontium content in the vegetative organs of IG measured after the treatment with ammonium metavanadate.

Sample	Sr (mg/kg)	V (mg/kg)
IG leaves 10 mg/L NH_4_VO_3_	38.2	2.7
IG roots 10 mg/L NH_4_VO_3_	37.3	624.4
IG rhizomes 10 mg/L NH_4_VO_3_	56.1	56.7
IG leaves 20 mg/L NH_4_VO_3_	43.4	8.2
IG roots 20 mg/L NH_4_VO_3_	37.6	590.8
IG rhizomes 20 mg/L NH_4_VO_3_	54.6	62.6
IG leaves 40 mg/L NH_4_VO_3_	44.3	11.1
IG roots 40 mg/L NH_4_VO_3_	50.0	921.4
IG rhizomes 40 mg/L NH_4_VO_3_	59.6	120.0
IG leaves control	45.0	0.7
IG roots control	38.4	54.6
IG rhizomes control	60.8	4.6

The growth responses varied with concentration. Leaf length (Fig. S13) and leaf number (Fig. S14) were not significantly affected at lower concentrations but showed reductions at 40 mg/L, with statistical significance reached for leaf length only. Leaf area (Fig. S15) and leaf dry mass (Fig. S16) decreased with increasing V concentration, with significant effects observed at the highest concentration in some of the models. Root, rhizome, and total biomass (Figs. S17–S19) showed decreasing trends, although these did not reach statistical significance. A summary of the growth responses is presented in Table 5.

**Table 5:** Summary of growth parameters (mean ± s.d.), and statistical significance for IG. Means within the same species followed by different letters are significantly different (*p* < 0.05).

Species	Treatment	Leaf length Mean ± s.d	Leaf number Mean ± s.d	Leaf area Mean ± s.d	Dry mass of leaves Mean ± s.d	Dry mass of roots Mean ± s.d	Dry mass of rhizome Mean ± s.d	Total dry mass Mean ± s.d
IG	Control	37.9 ± 11.1^a^	9.7 ± 2.3^a^	536.6 ± 352.6^a^	4.6 ± 2.9^a^	2.2 ± 2.0^a^	11.4 ± 9.2^a^	18.2 ± 13.9^a^
IG	V 10 mg/L	39.8 ± 8.6^a^	10.3 ± 0.8^a^	405.7 ± 134.5^a^	3.2 ± 0.5^a^	1.4 ± 0.7^a^	5.7 ± 2.7^a^	10.3 ± 3.6^a^
IG	V 20 mg/L	39.9 ± 12.1^a^	9.7 ± 1.9^a^	413.6 ± 127.2^a^	3.5 ± 1.4^a^	1.8 ± 0.6^a^	8.6 ± 3.5^a^	13.9 ± 5.2^a^
IG	V 40 mg/L	34.1 ± 9.2^b^	7.0 ± 2.1^b^	225.2 ± 117.1^a^	2.1 ± 1.0^a^	1.1 ± 0.8^a^	8.5 ± 5.0^a^	11.8 ± 6.6^a^
IG	Control	8.3 ± 5.6^a^	7.3 ± 1.8^a^	37.0 ± 15.6^a^	0.7 ± 0.3^a^	1.2 ± 0.5^a^	11.6 ± 5.5^a^	13.6 ± 6.0^a^
IG	Sr 50 mg/L	7.3 ± 4.6^a^	6.67 ± 2.2^a^	53.6 ± 28.4^a^	0.9 ± 0.4^a^	1.7 ± 0.6^a^	11.2 ± 7.2^a^	13.8 ± 7.9^a^
IG	Sr 100 mg/L	9.0 ± 7.2^a^	8.0 ± 0.6^a^	42.8 ± 32.3^a^	1.1 ± 0.4^a^	1.3 ± 0.6^a^	9.4 ± 5.7^a^	11.8 ± 6.6^a^
IG	Sr 200 mg/L	8.6 ± 6.6^a^	7.2 ± 1.5^a^	47.2 ± 20.9^a^	0.7 ± 0.2^a^	0.9 ± 0.5^a^	7.2 ± 4.8^a^	8.7 ± 5.1^a^

### The effect of strontium on *Iris × germanica* L. growth

Sr accumulated efficiently in all plant organs, with the highest levels observed in leaves, followed by roots and rhizomes (Table 4). Accumulation increased proportionally to the external concentration.

No statistically significant effects of Sr on morphological or biomass parameters were observed. Leaf characteristics (Figs. S20–S22) and the biomass parameters (Figs. S23–S26) remained unchanged across all the treatments (*p* > 0.05). The findings are summarized in Table 5.

## Discussion

To enable comparison with earlier phytoremediation research, all accumulation measurements in this study are presented as mg/kg dry weight (dw). When literature data are given in µg/g, these figures are numerically identical (1 µg/g = 1 mg/kg) and interpreted in the same way. Although there is no universally recognized threshold for hyperaccumulation of strontium or vanadium, hyperaccumulator plants are typically defined as species that can accumulate more than 1000 mg/kg (dw) of a specific metal in their aboveground parts, along with bioconcentration factor (BCF) and translocation factor (TF) values exceeding 1. These criteria serve as useful references for evaluating the accumulation capacity observed in this study Table 6.

**Table 6:** Strontium and vanadium content in the vegetative organs of IG measured after the strontium nitrate treatment.

Sample	Sr (mg/kg)	V (mg/kg)
IG leaves 50 mg/L Sr(NO_3_)_2_	439.1	2.2
IG roots 50 mg/L Sr(NO_3_)_2_	342.8	117.7
IG rhizomes 50 mg/L Sr(NO_3_)_2_	210.9	21.1
IG leaves 100 mg/L Sr(NO_3_)_2_	961.4	4.1
IG roots 100 mg/L Sr(NO_3_)_2_	776.7	179.1
IG rhizomes 100 mg/L Sr(NO_3_)_2_	310.5	45.6
IG leaves 200 mg/L Sr(NO_3_)_2_	1991.5	6.7
IG roots 200 mg/L Sr(NO_3_)_2_	1200.1	152.8
IG rhizomes 200 mg/L Sr(NO_3_)_2_	623.5	45.1
IG leaves control	28.4	10.0
IG roots control	33.0	26.8
IG rhizomes control	52.4	3.8

For a species to be considered a metal hyperaccumulator both TF and BCF must be > 1. These factors are measures of phytoextraction efficiency: TF is the absorbed metal shoot-to-root ratio, and BCF is the metals root-to-medium concentration ratio^41^. The TFs, BCFs, and TMU for the two species are listed in Table 7.

**Table 7:** TF, BCF, and TMU (mg/kg) for NE and IG for each V and Sr exposure.

V	TF	BCF	TMU
*NE*			
20 mg/L NH_4_VO_3_	0.04	65.24	1362.18
40 mg/L NH_4_VO_3_	0.03	61.48	2541.06
80 mg/L NH_4_VO_3_	0.05	23.81	2009.88
*IG*			
10 mg/L NH_4_VO_3_	0.00	62.44	683.82
20 mg/L NH_4_VO_3_	0.01	29.53	661.53
40 mg/L NH_4_VO_3_	0.01	23.03	1052.52
Sr	NE		
50 mg/L Sr(NO_3_)_2_	2.18	7.76	1234.61
100 mg/L Sr(NO_3_)_2_	1.98	6.64	1985.57
200 mg/L Sr(NO_3_)_2_	1.42	8.92	4333.93
*IG*			
50 mg/L Sr(NO_3_)_2_	1.28	6.85	992.80
100 mg/L Sr(NO_3_)_2_	1.23	7.76	2048.60
200 mg/L Sr(NO_3_)_2_	1.65	6.00	3815.10

In the case of vanadium, both species exhibited accumulation patterns consistent with previous reports, where V was predominantly retained in roots or underground organs, with limited translocation to shoots. NE concentrated more V in the underground than in the aboveground parts and was resistant to high doses of this heavy metal, which severely affected the growth of *Brassica juncea* L. and *Lycopersicon esculentum* L., wilting them in four days^42^. In our experiment, NE showed no signs of wilting after 14 days of treatment. Interestingly, the highest amount of V was accumulated by the plant rhizomes in contact with the 40 mg/L V solution, slightly more than that following the 80 mg/L V exposure. This ceiling effect can be explained by the fact that at 80 mg/L V, toxicity is so high that the cellular mechanisms of uptake and transport are inhibited, as oxidative stress is too high and cellular death occurs^43^. Although the cumulative concentration of V by NE was slightly lower than that of other species, NE exhibited remarkable tolerance to high vanadium levels. However, a notable drawback is the lower accumulation of vanadium in leaves compared to underground organs. Compared to the mean accumulated V values for *Chenopodium album* L. 637.9 mg/kg, *Kochia scoparia* (L.) Schrad 661.9 mg/kg, *Setaria viridis* (L.) P. Beauv 535.9 mg/kg^44^, NE in our study reached values exceeding 2000 mg/kg (dw) in rhizomes, indicating a comparatively high accumulation capacity, albeit largely restricted to underground organs. Neveretheless, the TF values remained well below 1, confirming limited translocation to the aerial parts. This behavior is consistent with the general classification of most plants as vanadium excluders or stabilizers rather than hyperaccumulators, supporting the conclusion that both species are more suitable for V phytostabilization than phytoextraction.

For Sr, both species demonstrated accumulation levels exceeding 1000 mg/kg (dw) in aerial tissues at higher exposure concentrations, reaching total plant concentrations of approximately 3800–4300 mg/kg (dw). These values are comparable to or higher than those reported for recognized Sr-accumulating species such as *Parthenocissus quinquefolia* (L.) Planch (~ 3900 mg/kg)^43^. In contrast to previous studies, where Sr is often preferentially retained in roots, both NE and IG showed efficient translocation to aerial organs (TF > 1). This is a key distinguishing feature, as effective phytoextraction requires both accumulation and translocation to harvestable biomass. Based on these criteria (high accumulation in shoots, TF > 1, and BCF > 1), both species meet the commonly used operational definitions of hyperaccumulator behavior for Sr, even though formal thresholds for this element remain less clearly established in the literature.

IG did not show signs of stress, necrosis, or visible growth inhibition in the presence of lower levels of V, but its growth was inhibited at the high 40 mg/L V dose. A concentration of 10 mg/L of ammonium metavanadate was found to be beneficial for enhancing the growth and development of rice^46^, and 20 mg/L V was beneficial for the growth and development of sweet corn^47^. IG exhibited good tolerance when grown in the presence of vanadium, but accumulated the metal in large amounts only within roots and not in rhizomes or leaves, quantities similar to those of *Chenopodium album* L., *Kochia scoparia* (L.) Schrad, *Setaria viridis* (L.) P. Beauv^44^. Based on these considerations, IG can be considered resistant, but not suitable for V phytoextraction, but rather for phytostabilization through root sequestration.

At all tested concentrations, IG demonstrated the highest Sr accumulation in leaves, followed by roots, with the lowest amount found in rhizomes. Despite metal exposure, plant growth remained unaffected, and no toxicity symptoms were observed in the present study. Previous studies on Sr-contaminated soil from silver mining in Turkey reported that Sr predominantly accumulates in underground plant tissues^45^. In contrast, IG accumulated a significantly higher Sr quantity than that reported in the cited study, with maximum accumulation in leaves. This pattern suggests IG’s potential utility in strontium phytoremediation, demonstrating remarkable translocation capabilities that qualify it as a potential hyperaccumulator. When exposed to 200 mg/L Sr, IG accumulated a total of 3,815.10 mg/kg throughout the entire plant—a quantity similar to the one accumulated by the established hyperaccumulator *Parthenocissus quinquefolia* (L.) Planch^43^.

This study has several limitations that must be considered when interpreting and contextualizing its findings. Our experiments were confined to a two-week hydroponic exposure under controlled hydroponic conditions; therefore, long-term field effects, interactions with soil chemistry or microbial communities, and seasonal effects have not been evaluated and require further study. Our research was only focused on vegetative growth endpoints; reproductive effects, secondary metabolite profiles, and oxidative stress potentially associated with the two metal toxicants were not assessed in this study. Future studies should focus on these endpoints. The doses used (≤ 200 mg L⁻^1^ Sr, ≤ 80 mg L⁻^1^ V) were based on similar ones published in the literature and are relevant for moderately contaminated sites, but may not be relevant for higher levels of contamination. The presence of V and Sr in the control plants was likely due to pre-existing metal stores accumulated before the study began. As hyperaccumulator species bind and store metals in the roots, rhizomes, and leaves through chelation and vacuolar sequestration, these internally retained ions persist even after two weeks of acclimation in a metal-free environment. Thus, the detected concentrations in the controls reflected residual internal metal pools rather than new uptake during the experiment. As each species was obtained from a single source, intraspecific genetic variation in metal uptake or the biological effects evaluated was not captured. Finally, we quantified the total amounts of the two metals, but not the metal speciation (e.g., Sr^2^⁺ vs. VO₄^3^⁻) and its influence on the uptake of the two, limiting the mechanistic interpretation of bioavailability patterns and biological effects.

The two evaluated species tolerated V and Sr ions very well without showing signs of chlorosis, wilting, or other signs of toxicity. Overall, when expressed in standardized units (mg/kg dw) and interpreted against commonly used phytoremediation benchmarks, our results position both species among the more efficient Sr-accumulating plants reported to date while confirming their limited applicability for vanadium phytoextraction. Both NE and IG can be used as Sr phytoremediation tools directly in soil or for rhizofiltration in hydroponically constructed water purification systems, depending on the atmospheric conditions, as NE requires more humidity and IG requires less. As they accumulate Sr in the aboveground organs, which regenerate, they can be harvested numerous times, and Sr can then be recovered.

## Supplementary Information

Below is the link to the electronic supplementary material.

Supplementary Material 1

## Data Availability

The datasets generated and/or analysed during the current study are available in the figshare repository, 10.6084/m9.figshare.31595119.
