# Supplementary material for: Iris × germanica L. and Nephrolepis exaltata (L.) Schott, two novel strontium hyperaccumulators
Source: Sci Rep. 2026 Jun 8;16:26159. doi: 10.1038/s41598-026-57010-6 (PMC13490416; doi:10.1038/s41598-026-57010-6)
Supplement: Supplementary file 1 [file 41598_2026_57010_MOESM1_ESM.docx]

**Supplementary Material**

**Table S1.** Atmospheric conditions for NE cultivated in the presence of ammonium metavanadate.

| **Grow tents** | **Average Temperature (^o^C)** | **S.D.** | **Average humidity (%)** | **S.D.** |
| --- | --- | --- | --- | --- |
| Grow tent #1 | 28.57 | 0.42 | 62.00 | 2.67 |
| Grow tent #2 | 29 | 0.67 | 61 | 2 |

**Table S2.** Nutrient solutions for NE cultivated in the presence of ammonium metavanadate.

| **Grow tanks** | **Average pH** | **S.D.** | **Average ppm** | **S.D.** | **Average Temperature (^o^C)** | **S.D.** |
| --- | --- | --- | --- | --- | --- | --- |
| Tank #1 | 6.10 | 0.48 | 471 | 43.33 | 28.56 | 0.43 |
| Tank #2 | 6.21 | 0.46 | 462 | 62 | 28.55 | 0.38 |
| Tank #3 | 6.12 | 0.45 | 522.67 | 100.22 | 28.85 | 0.52 |
| Tank #4 | 6.18 | 0.54 | 408.67 | 34.89 | 28.73 | 0.43 |

**Table S3.** Atmospheric conditions for NE cultivated in the presence of strontium nitrate.

| **Grow tents** | **Average Temperature (^o^C)** | **S.D.** | **Average humidity (%)** | **S.D.** |
| --- | --- | --- | --- | --- |
| Grow tent #1 | 25.4 | 0.67 | 64.67 | 13.78 |
| Grow tent #2 | 25.5 | 0.53 | 65.67 | 10.44 |

**Table S4.** Nutrient solutions for NE cultivated in the presence of strontium nitrate.

| **Grow tanks** | **Average pH** | **S.D.** | **Average ppm** | **S.D.** | **Average Temperature (^o^C)** | **S.D.** |
| --- | --- | --- | --- | --- | --- | --- |
| Tank #1 | 6.31 | 0.69 | 546.80 | 109.52 | 24.20 | 0.92 |
| Tank #2 | 6.23 | 0.85 | 447 | 229.20 | 22.95 | 2.84 |
| Tank #3 | 6.21 | 0.83 | 599.60 | 141.04 | 22.83 | 3.55 |
| Tank #4 | 6.03 | 0.99 | 551 | 129.20 | 22.72 | 3.64 |

**Table S5.** Atmospheric conditions for IG cultivated in the presence of ammonium metavanadate.

| **Grow tents** | **Average Temperature (^o^C)** | **S.D.** | **Average humidity (%)** | **S.D.** |
| --- | --- | --- | --- | --- |
| Grow tent #1 | 23.20 | 0.40 | 65.75 | 2.63 |
| Grow tent #2 | 23.53 | 0.48 | 63.25 | 2.25 |

**Table S6.** Nutrient solutions for IG cultivated in the presence of ammonium metavanadate.

| **Grow tanks** | **Average pH** | **S.D.** | **Average ppm** | **S.D.** | **Average Temperature (^o^C)** | **S.D.** |
| --- | --- | --- | --- | --- | --- | --- |
| Tank #1 | 5.36 | 0.61 | 638.80 | 24.56 | 23.25 | 0.37 |
| Tank #2 | 5.75 | 0.64 | 645.25 | 22.75 | 23.28 | 0.42 |
| Tank #3 | 5.45 | 0.65 | 664.20 | 10.56 | 23.84 | 0.43 |
| Tank #4 | 4.34 | 0.79 | 624.80 | 20.16 | 23.60 | 0.40 |

**Table S7.** Atmospheric conditions for IG cultivated in the presence of strontium nitrate.

| **Grow tents** | **Average Temperature (^o^C)** | **S.D.** | **Average humidity (%)** | **S.D.** |
| --- | --- | --- | --- | --- |
| Grow tent #1 | 24.33 | 0.28 | 49.00 | 1.50 |
| Grow tent #2 | 24.23 | 0.33 | 47.50 | 1.50 |

**Table S8.** Nutrient solutions for IG cultivated in the presence of strontium nitrate.

| **Grow tanks** | **Average pH** | **S.D.** | **Average ppm** | **S.D.** | **Average Temperature (^o^C)** | **S.D.** |
| --- | --- | --- | --- | --- | --- | --- |
| Tank #1 | 4.89 | 1.22 | 665.67 | 17.78 | 23.12 | 0.07 |
| Tank #2 | 5.03 | 1.26 | 671.67 | 19.78 | 23.10 | 0.08 |
| Tank #3 | 5.04 | 1.32 | 648.00 | 16.67 | 23.57 | 0.10 |
| Tank #4 | 4.93 | 1.18 | 670.33 | 9.78 | 23.50 | 0.10 |

**Figure S1.** Variation in leaf length of NE as a function of the concentration of vanadium

**Figure S2.** Variation in leaf number in NE as a function of vanadium ion concentration

**Figure S3.** Leaf area variation in NE as a function of the vanadium ion concentration

**Figure S4.** Variation in leaf dry mass of NE as a function of vanadium ion concentration

**Figure S5.** Change in the dry mass of the underground organs of NE as a function of the vanadium ion concentration

**Figure S6.** Variation of total dry mass of NE with vanadium ion concentration

**Figure S7.** Variation in leaf length of NE as a function of strontium ion concentration

**Figure S8.** Variation in leaf number of NE as a function of strontium ion concentration

**Figure S9.** Leaf area variation in NE as a function of strontium ion concentration

**Figure S10.** Variation in leaf dry mass of NE as a function of strontium ion concentration

**Figure S11.** Variation in dry mass of underground organs in NE as a function of strontium ion concentration

**Figure S12.** Variation of total dry mass of NE as a function of strontium ion concentration

**Figure S13.** Leaf length variation of IG under the influence of ammonium metavanadate treatment

**Figure S14.** Leaf number variation in IG under the influence of ammonium metavanadate treatment

**Figure S15.** Leaf area variation in IG under the influence of ammonium metavanadate treatment

**Figure S16.** Leaf dry mass variation of IG under the influence of ammonium metavanadate treatment

**Figure S17.** Variation in the root dry mass of IG under the influence of ammonium metavanadate treatment

**Figure S18.** Variation in the rhizome dry mass of IG under the influence of ammonium metavanadate treatment

**Figure S19.** Variation in the total dry mass of IG under the influence of ammonium metavanadate treatment

**Figure S20.** Leaf length variation of IG under the influence of strontium nitrate treatment

**Figure S21.** Leaf number variation in IG under the influence of strontium nitrate treatment

**Figure S22.** Leaf area variation of IG under the influence of strontium nitrate treatment

**Figure S23.** Leaf dry mass variation of IG under the influence of strontium nitrate treatment

**Figure S24.** Variation in root dry mass of IG under the influence of strontium nitrate treatment

**Figure S25.** Changes in the dry rhizome mass of IG under the influence of strontium nitrate treatment

**Figure S26.** Change in total dry mass of IG under the influence of strontium nitrate treatment
